# Characteristics of Microparticles Based on Resorbable Polyhydroxyalkanoates Loaded with Antibacterial and Cytostatic Drugs

**DOI:** 10.3390/ijms241914983

**Published:** 2023-10-07

**Authors:** Anastasiya V. Murueva, Anna M. Shershneva, Ekaterina I. Shishatskaya, Tatiana G. Volova

**Affiliations:** 1 Institute of Biophysics SB RAS, Federal Research Center “Krasnoyarsk Science Center SB RAS” (IBP SB RAS), 50/50 Akademgorodok, 660036 Krasnoyarsk, Russia; goreva_a@mail.ru (A.V.M.); shishatskaya@inbox.ru (E.I.S.); 2 Institute of Fundamental Biology and Biotechnology, Siberian Federal University, 79 Svobodny Pr., 660041 Krasnoyarsk, Russia; anytka712@mail.ru; 3 Chemistry Engineering Centre, ITMO University, Kronverkskiy Prospekt, 49A, 197101 Saint Petersburg, Russia

**Keywords:** drug delivery systems, biodegradable polyhydroxyalkanoates, microparticles, properties, drug efficiency, *E. coli* and *HeLa* cultures

## Abstract

The development of controlled drug delivery systems, in the form of microparticles, is an important area of experimental pharmacology. The success of the design and the quality of the obtained microparticles are determined by the method of manufacture and the properties of the material used as a carrier. The goal is to obtain and characterize microparticles depending on their method of preparation, the chemical composition of the polymer and the load of the drugs. To obtain microparticles, four types of degradable PHAs, differing in their chemical compositions, degrees of crystallinity, molecular weights and temperature characteristics, were used (poly-3-hydroxybutyrate and copolymers 3-hydroxybutyric-co-3-hydroxyvaleric acid, 3-hydroxybutyric-co-4-hydroxybutyric acid, and 3-hydroxybutyric-co-3-hydroxyhexanoic acid). The characteristics of microparticles from PHAs were studied. Good-quality particles with an average particle diameter from 0.8 to 65.0 μm, having satisfactory ζ potential values (from −18 to −50 mV), were obtained. The drug loading content, encapsulation efficiency and in vitro release were characterized. Composite microparticles based on PHAs with additives of polyethylene glycol and polylactide-co-glycolide, and loaded with ceftriaxone and 5-fluorouracil, showed antibacterial and antitumor effects in *E. coli* and *HeLa* cultures. The results indicate the high potential of PHAs for the design of modern and efficient drug delivery systems.

## 1. Introduction

Designing new drug formulations is a priority task in biomedicine and experimental pharmacology. New-generation drug delivery systems (DDS) enable the prolonged release of the drugs, enhance their bioavailability, direct them to the site of a pathological process, and reduce the possible side effects of the drugs. Modern research is focused on the development of long-term and targeted dosage forms of micro- and nanosized particles based on various materials, the use of which makes it possible to influence the properties of the drugs, including the in vivo route of administration, biodistribution, permeability and pharmacokinetics [1,2,3,4,5,6]. An ideal DDS should be inert, biocompatible, mechanically strong and stable, provide high bioavailability to the drug and protection from accidental release, be easy to administer, be able to deliver the drug to the target site, and be therapeutically effective, easy to manufacture and compatible with a wide range of drugs. DDSs have a number of advantages over free drugs: the drugs are retained in the patient’s system for longer periods of time; they have better pharmacokinetics, and many of the drugs become able to cross the membrane and blood–brain barriers; hydrophobic drugs become soluble; and possible side effects of highly toxic drugs are reduced [7,8,9,10,11,12,13,14]. New DDSs protect drugs from degradation and reduce initial drug concentrations and the number of administrations in the case of long-term medication [6,15,16,17,18,19].

The most widely used materials for constructing DDSs are polyesters such as poly(ε-caprolactone) (PCL), poly(lactic acid) (PLA), poly(lactide-co-glycolide) (PLGA) [20,21], polyanhydrides [22,23,24], poly(orthoesters) [25,26]; nonionic surfactants [4]; polysaccharides (starch, dextran, chitosan) [27,28,29,30]; and, in recent years, polyhydroxyalkanoates (PHAs).

PHAs are a class of biocompatible and biodegradable thermoplastics with different physicochemical structures and properties [31,32,33,34,35,36,37,38,39,40]. The chemical and physical properties of the material depend on the monomer component of PHAs. Because of their various structural and mechanical properties, PHAs are being employed in many technological fields [41]. They can be processed into various products using almost all available technical methods (casting, emulsification, extrusion, pressing, electrostatic molding, etc.) [42,43]. The areas of application of PHAs are diverse, from agriculture and the urban economy to high-tech biomedicine and pharmaceutical production [44,45,46,47], and they also play a significant role in the “circular economy” [48]. The excellent biocompatibility of PHAs, and their long-term and directed resorption in vivo, affirms this class of biopolymers as the most promising for medical bioengineering. The biocompatibility characteristic of PHA polymers is due to (3-hydroxybutyric acid), which is produced by cell metabolism and exists in human blood [49]. Moreover, the constant local pH value during degradation allows PHAs to be highly compatible with cells and the immune system in comparison with other popular polymers, such as PCL, PLA and PLGA [50]. Due to this, PHAs are especially promising in personalized medicine, cell technologies and tissue engineering, as well as modern pharmacology and drug delivery [31,32,51,52,53,54,55,56]. The most-studied member of the PHA family is poly-3-hydroxybutyrate P(3HB); however, due to its high melting point, low plasticity, and high crystallinity, approaches are being developed to obtain micro- and nanoparticles using copolymers with 3-hydroxybutyric-co-3-hydroxyvaleric acid P(3HB-co-3HV), 3-hydroxybutyric-co-4-hydroxybutyric acid P(3HB-co-4HB), and 3-hydroxybutyric-co-3-hydroxyhexanoic acid P(3HB-co-3HHx), and also composites with other biocompatible materials [57,58].

The most popular method for obtaining micro- and nanoparticles is the emulsion method (solvent evaporation method). Using this method, PHA micro- and nanoparticles loaded with various drugs (antibiotics, anesthetics, steroids, hormones, anticancer agents, anti-inflammatory agents, and vaccines) have been obtained [32,41,58,59,60].

Among the different techniques used, the spray-drying method is a simple, economical and easily scalable process to produce microspheres or microcapsules for encapsulating various biologically active substances in the pharmaceutical, chemical, cosmetic, and food industries [61,62,63,64,65]. One of the main advantages of obtaining microparticles from polyesters using spray drying is the use of solvents with a low boiling point. So, the use of dichloromethane or chloroform prevents the agglomeration of microparticles [66]. Another advantage of spray drying is that the droplets are exposed to temperatures for milliseconds, which protects the active compounds from thermal degradation. Moreover, this method allows one to obtain particles with high drug-encapsulation efficiency, in contrast to the emulsion method, in which the drug is separated between the phases in the emulsion; as a result, this leads to a decrease in the above indicator [67,68].

Using the spray-drying method, microparticles containing various antitumor, anti-inflammatory and antibacterial drugs have been obtained [69,70,71]. The most popular materials for obtaining microparticles using spray drying are chitosan, gelatin, starch, and hyaluronic acid, as well as PCL, PLA, and PLGA [72,73].

Despite the promise of this method in relation to PHA, there have been isolated examples of obtaining microparticles loaded with drugs mainly from P(3HB), for example, containing paracetamol and paclitaxel [74,75]. At the same time, there are very little data on the effect of the chemical composition of PHAs and the parameters of the spray-drying process on the properties of microparticles (size, zeta potency, drug encapsulation efficiency).

In Russia, the study of PHAs for drug deposition and delivery was started at the Institute of Biophysics of the Siberian Branch of the Russian Academy of Sciences in 2008 using P(3HB) and one type of PHAs copolymer—P(3HB-co-3HV)—with the inclusion of 3HV monomers not higher than 11 mol.%. Microparticles were prepared from two-component and three-component emulsions [76,77]. Particular attention has been paid to the study of the safety and biocompatibility of microparticles, which has been performed in vitro and in vivo [78,79].

Despite active research and a fairly large amount of information obtained on the development of a DDS based on PHAs, which has been actively carried out by a large number of research teams in many countries, many key issues need to be comprehensively studied. First of all, studies on the use of PHAs with a different set and ratio of monomers for the design of DDSs, which have various and improved technological properties compared to the most-studied P(3HB), are of current interest. Most of the known works are limited to the use of an emulsion method for obtaining PHA micro- and nanoparticles, without taking into account the effect of the chemical composition of the polymer on the properties of polymer carriers. Data on the possibility of using the spray-drying process for the deposition of drugs in PHA microparticles, including in compositions with other biopolymers, are extremely limited.

This has determined the goal of this work—to investigate the properties of microparticles depending on the method and parameters of production, the chemical composition of a polymer base from PHA, and the loads of drugs with different mechanisms of action.

## 2. Results

### 2.1. Influence of Manufacturing Methods on the Properties of P(3HB) Microparticles

The characteristics of PHA microparticles obtained using the emulsion method and the spray-drying method were studied. The development and study of these methods was carried out on the example P(3HB), as the most accessible and studied type of polymer from the PHA family. The quality assessment of the resulting polymer microparticles included the registration of the morphology, size distribution, mean diameter, and ζ potential; the value of the yield of the obtained microparticles served as an indicator of the performance of the technology.

**Figure 1:**
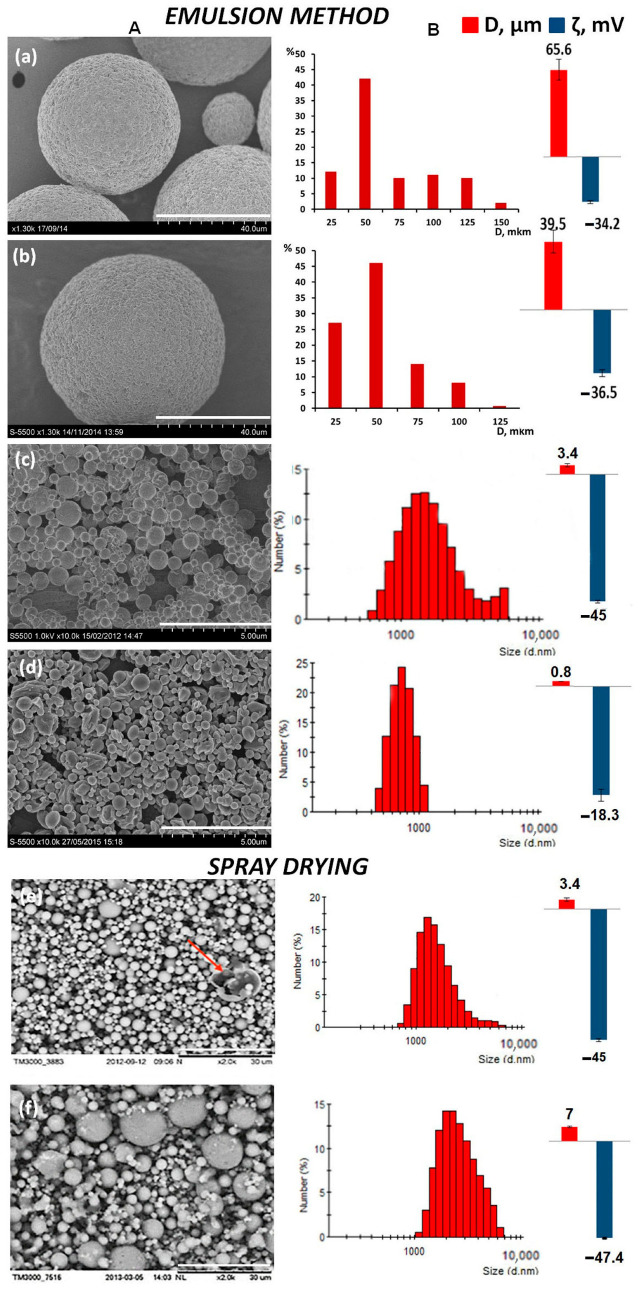
Characteristics of microparticles from P(3HB) obtained using various methods: (**A**)—SEM image of microparticles; (**B**) size distribution, mean diameter (μm) and ζ potential (mV) of microparticles. Emulsion was mixed at speeds of 500 rpm (**a**), 1000 rpm (**b**), 15,000 rpm (**c**), and 24,000 rpm (**d**); spray-drying process parameters were as follows: feed pump speed and temperature 75 °C, 1.5 mL/min (**e**), 95 °C, 3.2 mL/min (**f**); the red arrow indicates the burst particle. The scale bar is 40 μm (**a**,**b**), 5 μm (**c**,**d**), and 30 μm (**e**,**f**).

The characteristics of microparticles are presented in Figure 1 and in Table 1. A double emulsion containing a solution of P(3HB) and PVA was used. When developing this method, the concentration of the polymer in the emulsion, the stirring rate, and the method of dispersion were varied. When studying the effect of the rate and method of micronization of the polymer emulsion, a 2% (*w*/*v*) P(3HB) solution and 0.5% (*w*/*v*) PVA were used. Regardless of the speed mode of mixing the emulsion with mixers, all particles were of a correct spherical shape, but were heterogeneous in size, and their average diameter varied by orders of magnitude from 25 to 100 μm and above. The stirring rate significantly affected the size of the microparticles. With an increase in the stirring speed from 500 to 1000 rpm, the average diameter of the microparticles decreased by 1.5 times, from 65.6 ± 7.8 to 39.5 ± 9.2 μm; the yield of microparticles was close −68.4 ± 3.6% and 72 ± 4.6% at a speed of 500 rpm and 1000 rpm, respectively. At higher mixing speeds, the particle size was significantly reduced. The particle sizes obtained at emulsion micronization rates of 15,000 and 24,000 rpm had an average diameter of 1.58 ± 0.15 and 0.8 ± 0.12 μm, respectively.

The mixing speed of a polymer emulsion is influenced by another important parameter, the microparticle ζ potential, which characterizes the stability or coagulation of microparticles in a dispersion medium [80,81,82]. The microparticles from P(3HB) produced using the emulsion method had satisfactory ζ potential values (from −36.5 mV to −15 mV). At the same time, large particles (65 and 40 microns) had more-negative ζ potential values ζ potential (Figure 1a,b). The effect of different concentrations of the polymer solution (1.0, 2.0 or 4.0% (*w*/*v*)) on particle characteristics was studied. The process of obtaining particles was carried out at an emulsion stirring speed of 15,000 rpm. The results presented in Table 1 indicate the influence of the concentration of the polymer solution, and the type of micronization of the emulsion, on the characteristics of the particles.

When stirring the emulsion with a high-speed stirrer at 15,000 rpm, the most-concentrated polymer solution produced particles of the largest diameter (2.32 ± 0.3 μm), which was comparable to the particle size obtained using the 12 V power ultrasound. The diameter of particles obtained from less-concentrated P(3HB) solutions (1% and 2% (*w*/*v*)) were 1.2 ± 0.09 and 1.58 ± 0.15 μm, respectively; a similar result was obtained through the micronization of the emulsion with a higher-power ultrasound (20 V). The use of more-concentrated and more-viscous P(3HB) solutions reduces the efficiency of mixing and crushing droplets into smaller particles, increasing their size. This effect has been described before [83,84,85]. The influence of the concentration of the polymer in the emulsion and the sonication power on the ζ potential of the microparticles was reversed: the lowest-charged particles (−21.6 ± 0.7 mV) were obtained from the most-concentrated solution and at the lower sonication power of the emulsion (−23 ± 0.31 mV). In addition, the use of ultrasound reduced the yield of microspheres by an average of 20% compared with the yields of particles obtained by mixing the emulsion with a stirrer and a high-speed homogenizer. In general, varying the emulsion micronization conditions and using one type of PHA as an example showed the possibility of obtaining microparticles with a wide size range of diameters, including particles of less than 1.0 μm, which are the most acceptable in systemic drug delivery. This type of delivery, usually via the intravenous route, generally favors smaller particles to avoid their rapid and/or non-specific clearance from the blood stream, and embolic phenomena [86,87]. (In contrast, microspheres with a size above about 4–10 μm or more are mechanically trapped and filtered out by the first capillary bed after intravenous injection, but can be used in local drug delivery [86,88]).

When using the Buchi B-290 Spray dryer to obtain P(3HB) microparticles, it turned out that it did not allow one to obtain good-quality particles from polymer solutions with high molecular weights. As a result, instead of microparticles, “polymer filaments” are formed, and the yield of the product in the receiving cyclone does not exceed 10%. However, the use of P(3HB) samples with a molecular weight of less than 100 kDa, which were obtained using borohydride as a depolymerizing agent, facilitated the formation of good-quality spherical particles at high indicators of the yield of microparticles. In the process of developing the method, a 1% (*w*/*v*) solution of P(3HB) was used; the feed pump speed varied from 1.0 to 5.0 mL/min. The variation in the solution feed rate was combined with changes in the temperature, which was 75, 85 or 95 °C (Figure 1, Table 1).

All the batches of microparticles obtained had a regular spherical shape and were heterogeneous in size, similar to the emulsion method; the particle sizes varied from 0.6 to 10 μm. In general, in all of the studied modes, regardless of the solution feed rate and temperature, the obtained microparticles, in comparison with the emulsion method, generally had closer values to the average diameter and electrokinetic potential. The smallest particles (mean diameter of 3.4 ± 0.6 and 3.8 ± 0.5 μm) were obtained at the lowest temperature and feed rate of the P(3HB) solution, respectively, of 75 °C and 1.5 and 3.2 mL/min; the largest ones (6.2 and 7.0 μm) were observed at 95 °C and at a higher solution flow rate (5.0 and 3.2 mL/min). In all the other variants, the particle size was close, about 5.0 μm. It was shown that the P(3HB) microparticles obtained using this method were hollow, but could contain smaller particles in their internal cavity. In the photo of the microparticles (75 °C, solution flow rate of 1.5 mL/min), an arrow shows a large broken particle with an empty internal cavity (Figure 1e). The presence of deformed and broken particles in almost all variants of obtaining should be noted, but their share did not exceed 1.0% in the total yield of microparticles. The value of the ζ potential of the microparticles was also comparable, and varied from −41 to −50.8 mV (Table 1). The yield of microparticles obtained using spray drying varied from 45 to 90%.

### 2.2. Influence of the Chemical Composition of PHAs on the Properties of Microparticles

**Figure 2:**
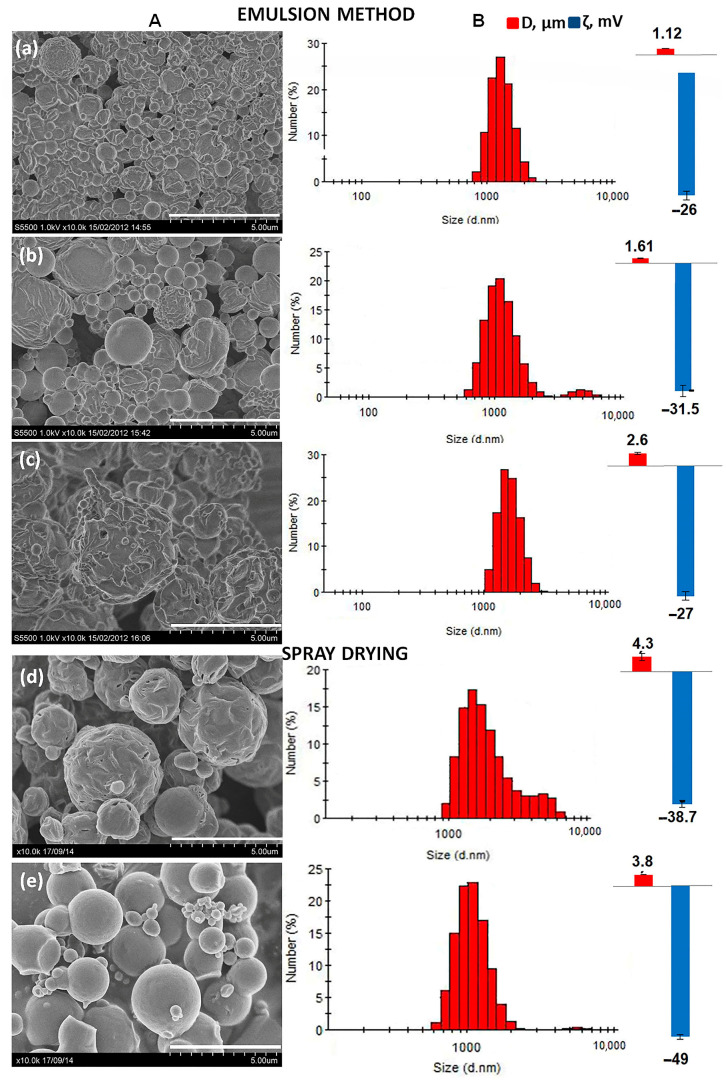
Characteristics of PHA microparticles of various chemical compositions and in compositions with PEG and PLGA: (**A**) SEM image of microparticles; (**B**) size distribution, mean diameter (μm) and ζ potential of microparticles (mV). The chemical compositions of microparticles as follows: P(3HB/3HV) 93.2/6.8 (**a**), P(3HB/4HHx) 86.4/13.6 (**b**), P(3HB/4HB) 84.0/16.0 (**c**), P3HB/PEG 75/25 (**d**), and P3HB/PLGA 75/25 (**e**). The scale bar is 5 μm.

To reveal the effect of the actual chemical composition of PHA samples with different properties, using the emulsion method, a series of microparticles was obtained from three types of copolymers with different ratios of monomers (Table 1). An emulsion was used containing a 2% (*w*/*v*) polymer solution and a 0.5% (*w*/*v*) PVA solution, with a stirring speed of 24,000 rpm. All batches of the obtained microparticles were heterogeneous in size (the diameter of different particle fractions varied in the range of 0.4 to 6 μm), while differences in the particle characteristics were revealed, including sizes and morphology. Figure 2 shows the characteristics of PHA microparticles obtained from copolymers with the highest contents of 3-HV (32.8 mol.%), 3-HHx (13.6 mol.%) and 4-HB (16.0 mol.%) monomers.

The microparticles obtained from the P(3HB-co-3HV) copolymers (6.8 mol.% of 3HV monomers) had an average diameter of 0.76 ± 0.09 μm, and were of a regular rounded shape. The particles obtained from the same copolymer but with a significantly higher content of 3HV monomers (32.8 mol.%) had an average diameter of about 1.12 ± 0.17 μm. In addition, the surface of the microparticles was embossed. Among the harvested microparticles, the presence of particles of irregular shape was observed. The microparticles obtained from P(3HB-co-4HB) were larger. The microparticles obtained from the copolymers P(3HB-co-3HHx) (7.0 and 13.6 mol.% of 3HHx monomers) and P(3HB-co-4HB) (6.1 mol.% of 4HB monomers) had a spherical shape with a smooth surface. The average diameter of the microparticles obtained from P(3HB-co-3HHx) was comparable to the particles from P(3HB-co-3HV) (32.8 mol.% content of 3-HV; their average diameter was 1.14 ± 0.15 μm and 1.61 ± 0.05 μm (7.0 and 13.6 mol.% of 3HHx monomers)). The sizes of the microparticles obtained from the P(3HB-co-4HB) copolymer were significantly higher compared to those of the other copolymer microparticles (2.3 ± 0.16 and 2.6 ± 0.24 μm), with the inclusion of monomers of 4HB of 6.1 mol.% and 16.0 mol.%, respectively. In general, the presence of 3-HV, 3-HHx and 4-HB monomers in PHA was accompanied by an increase in the particle size compared to particles from a homopolymer obtained under similar conditions.

An addition to the effects on the particle sizes, it was shown that the monomer composition of the polymer had a significant effect on the ζ potential of the particles. The microparticles from P(3HB-co-3HHx) and P(3HB-co-4HB) had the closest and lowest values of the ζ potential (−31.5 mV, and −29 and −27 mV (16 mol.% and 6.1 mol.% of 4HB monomers), respectively. The microparticles from P(3HB-co-3HV) had a close ζ-potential, in the order of −23 and −26 mV depending on the content of the 3HV monomers in copolymers. The microparticles from the homopolymer had a ζ potential of −18.3 mV. The particles obtained from copolymeric PHAs had lower values of the ζ potential compared to the P(3HB) homopolymer, which indicates their higher physical stability.

P(3HB) is a highly crystalline polymer; therefore, various approaches for changing its physicochemical properties were studied in this work. One approach is to obtain composites with other biocompatible but low-crystalline materials. Microparticles from the mixture of P(3HB)/PLGA and P(3HB)/PEG polymers were obtained using the spray-drying method. It was shown that the addition of low-molecular-weight and hydrophilic PEG contributed to the formation of a rough particle structure and pore formation. Microparticles prepared from composite P(3HB)/PLGA were of a spherical shape, without deformations and pores. The average diameter of composite microparticles obtained from a mixture of P(3HB)/PLGA and P(3HB)/PEG was comparable and amounted to 3.8 ± 0.6 μm and 4.3 ± 0.8 μm, respectively. Composite microparticles P(3HB)/PLGA (−49 mV) had the most negative zeta potential values. This is apparently due to an increase in the number of negatively charged carboxyl groups in the composite. Thus, the addition of low-molecular-weight polymers to P(3HB) makes it possible to influence the surface structure of microparticles, size characteristics, and values of the zeta potential of microparticles.

### 2.3. Drug-Loaded PHA-Microparticle and Drug Release In Vitro

Well-established methods for obtaining good-quality microparticles have made it possible to start designing dosage forms loaded with drugs, and studying the dynamics of their release in vitro depending on the method of obtaining microparticles, the type and degree of drug loading, and the monomer composition of the PHAs.

The choice of the drugs was based on their demand in clinical practice for the treatment of long-lasting diseases, their stability in solution, and the feasibility of mixing the drug with no polar solvents without any change in the properties of the drugs.

In earlier work, we investigated the possibility of the deposition of doxorubicin into microparticles from P(3HB) using the emulsion method and studied the properties and medicinal efficacy of the microparticles [89]. In continuation of the topic, in this work, in order to compare, the effect of the monomeric composition of PHAs on the properties of microparticles loaded with the same cytostatic, and the drug release in vitro, was studied.

**Figure 3:**
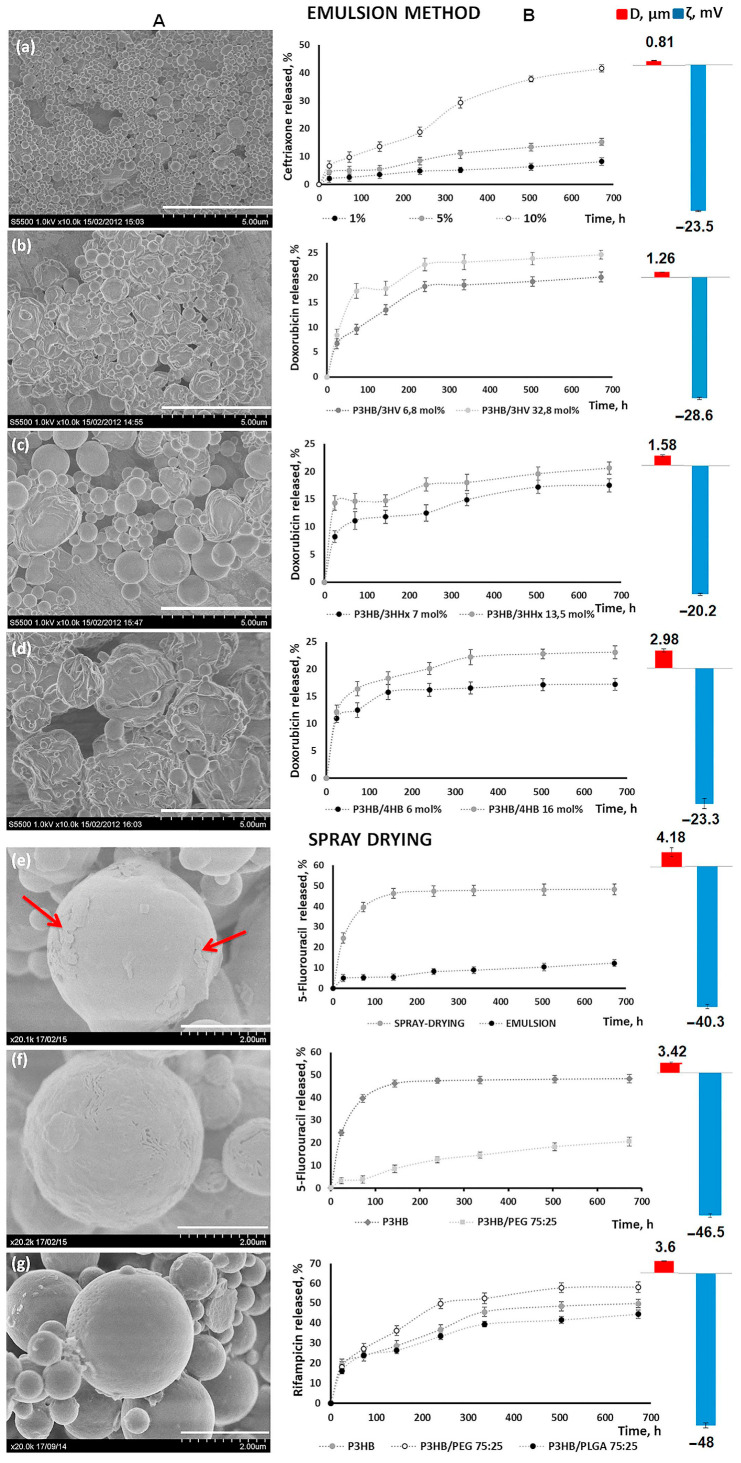
Characteristics of drug-loaded microparticles from PHAs and in composition with PEG and PLGA obtained using the emulsion method and spray-drying method: (**A**) SEM image of microparticles; (**B**) drug release from PHAs microparticles, and average diameter (μm) and ζ potential (mV) of microparticles. SEM image, ζ potential and average diameter are given for P3HB microparticles containing 10% ceftriaxone (**a**), P(3HB/3HV) 67.2/32.8 containing 5% doxorubicin (**b**), P(3HB/4HHx) 86.4/13.6 containing 5% doxorubicin (**c**), P(3HB/4HB) 84.0/16.0 containing 5% doxorubicin (**d**), P3HB microparticles containing 5% 5-fluorourocil (**e**); the red arrow indicates the drug, P3HB/PEG 75/25 containing 5% 5-fluorourocil (**f**), and P3HB/PLGA 75/25 containing 5% rifampicin (**g**). The scale bar is 5 μm (**a**–**d**) and 2 μm (**e**–**g**).

Doxorubicin is a cytotoxic anthracycline antibiotic isolated from a culture of *Streptomyces peucetius*. The chemical compositions of the PHAs influenced the release of this drug in vitro (Figure 3b–d).

Microparticles from P(3HB-co-3HV) with a low content of 3HVmonomers (6.8 mol.%) had a smooth surface and a regular spherical shape. The surface of microparticles obtained from other types of copolymer PHAs—from medium-chain P(3HB-co-3HHx), as well as from the elastic copolymer P(3HB-co-4HB)—had a relief structure with irregularly shaped particles, but their amounts did not exceed 10% in the particle yield. The average diameter of the microparticles varied from 1.26 to 2.98 μm; the values of the zeta potential were from −20.2 to −28.6 mV, depending on the monomeric composition of the polymer (Figure 3).

The efficiency of doxorubicin encapsulation was quite high, and varied slightly depending on the PHAs’ monomer compositions, ranging from 63 to 77%. The highest values of the encapsulation efficiency of doxorubicin are typical for microparticles obtained from copolymers P(3HB-co-3HHx) and P(3HB-co-4HB): 75.2 ± 7.2% and 77.4 ± 7.7%, respectively.

The surface of microparticles from P(3HB-co-3HV), P(3HB-co-3HHx), and P(3HB-co-4HB) copolymers loaded with doxorubicin was generally similar to the unloaded particles from these copolymers. Figure 3c shows doxorubicin release curves from microparticles obtained from the PHAs of various chemical compositions. On the first day, an active release of the drug was noted, up to 7.7 ± 1.2% and 9.1 ± 1.3% for microparticles with a content of 3HV monomers of 6.8 and 32.8 mol.%, respectively. Further, this trend persisted for particles with a high content of 3HV monomers, and after 3 days (72 h), the drug yield was 16.8 ± 1.1%.

This was slightly higher than that of the particles with a low content of 3HHx, the doxorubicin outflow of which after 3 days was 13.7 ± 1.4%. Starting from the 10th day (240 h) of the experiment, the yield of the drug slowed down, and by the end of the observation it was about 20 and 24% for the microparticles from P(3HB-co-3HV) (the content of the 3HB monomers was 6.8 and 32.8 mol.%., respectively).

The doxorubicin release profiles from microparticles based on a P(3HB-co-3HHx) with the inclusion of 3HHx monomers (7.0 and 13.6 mol.%) also showed an active release of doxorubicin on the first day. It was shown that the release of doxorubicin increased with an increase of the 3HHx content in the copolymer. During the first day of observation, the proportion of released doxorubicin was 8.5 and 13.8% for samples with the content of 3HHx 7.0 and 13.6 mol.%, respectively. In the case of the microparticles with the inclusion of 3HHx 13.6 mol.%, after a sharp increase to 13.8%, the concentration of the released drug remained at this level up to 6 days. By the end of the experiment, the cumulative drug release was 20.6%.

Figure 3d shows the drug release profile of doxorubicin-loaded microparticles prepared from the P(3HB-co-4HB) copolymer, with a 4HB content of 6.1 and 16 mol.%. It was shown that nearly 10% doxorubicin was released in the first 24 h. The total drug release by the end of the experiment was 17.2 and 23.1% for the P(3HB-co-4HB) microparticles with the studied level of 4HB monomers.

In the study of microparticles loaded with doxorubicin and obtained from the P(3HB-co-4HB) copolymer, with the inclusion of 4HB monomers of 6.1 and 16 mol.%, the doxorubicin release on the first day was 10.4 and 12.1%, respectively (Figure 3d).

Thus, the release of doxorubicin from the obtained PHA microparticles depended on the monomer composition of the PHAs, and increased with an increase in the contents of the 3HV, 3HHx, and 4HB monomers. The increase in doxorubicin release from PHA copolymers may be due to the physicochemical properties of the material. As a rule, with an increase in the content of monomers such as 3HB, 4HB, 3HHx, a decrease in the molecular weight and degree of crystallinity of the polymer occurs (Table 2). It has been shown that the drug release may depend on the molecular weight and crystallinity of the polymers [90]. Thus, the diffusion of drugs occurs faster from the amorphous regions of the polymer, while the drug bound to the crystalline regions of the polymer is more tightly bound, and diffusion can be difficult [91,92]. Due to the fact that an increase in the monomers 3HB, 4HB, 3HHx leads to a decrease in the degree of crystallinity of the polymer, the rate of doxorubicin outflow may increase.

The study of the influence of the content of a drug on the characteristics of microparticles was investigated on the model antibacterial drug ceftriaxone. P(3HB) was chosen as the polymeric material because submicron particles are considered to be more desirable in the development of DDSs. As described above, the average diameter of the P(3HB) microparticles was 0.8 µm or less.

To study the effect of this parameter on the yield of the drug, using the method of solvent evaporation, polymeric microparticles based on P(3HB) were obtained from a three-component emulsion (2% (*w*/*v*) solution of P(3HB) in DCM, 0.5% (*w*/*v*) PVA, and emulsion stirring speed 24,000 rpm) with different contents of ceftriaxone. All the samples of microparticles loaded with this drug (Figure 3a) had a spherical regular shape. With an increase in the amount of ceftriaxone in the particles (1.0. 5.0 and 10.0% by the weight of the polymer), a change in their average diameters was noted, respectively, up to 0.812 μm, and there was a change in the zeta-potential value to −23.5 mV.

With an increase in the content of the drug in microparticles, the efficiency of its encapsulation decreased, amounting to 81.5 ± 3; 74.6 ± 4.9 and 62.3 ± 6.5%, respectively, at the achieved particle load. On day 3, the proportion of released ceftriaxone was low and amounted to 2.6 ± 0.4, 5.1 ± 0.3 and 9.3 ± 0.5% for the microparticles loaded with the drug by 1.0, 5.0 and 10.0%, respectively. By the end of the observation, the proportion of the drug released from that included in the microparticles was 8.2 ± 1.2, 15.2 ± 1.1 and 41.6 ± 1.3% (Figure 3a). In general, the release of the antibiotic from the P(3HB) microparticles was smooth during the entire experiment and was without sharp outbursts, and depended on the loading of the particles with this drug.

To study the influence of the manufacturing method on the characteristics of the microparticles, P(3HB) and the antitumor drug 5-fluorourocil were used as the polymeric material. The choice of P(3HB) as a polymer carrier was due to the fact that, as a polymeric material with a molecular weight of less than 100 kDa, it is suitable for obtaining microparticles using spray drying. As described above, the depolymerization procedure was successfully implemented only for P(3HB). It was not possible to reduce the molecular weight of the PHA copolymers to the required values.

The results regarding the influence of the method of obtaining microparticles loaded with 5-fluorourocil on their characteristics and the release of the drug are shown in Figure 3. The average diameter of the microparticles loaded with 5-fluorourocil and obtained using the emulsion method with 2% (*w*/*v*) P(3HB) solution, at an emulsion stirring speed of 24,000 rpm), was 0.87 ± 0.22 μm, with a ζ potential of −19.4 ± 0.61 mV.

Particles similar in composition obtained using spray drying (a solution feed rate of 1.5 mL/min, 75 °C) had an average diameter of 4.18 ± 0.15 μm and a ζ potential of −40.3 ± 0.75 mV. The efficiency of the encapsulation of 5-fluorourocil in microparticles obtained using spray drying was two times higher than the emulsion method, and amounted to 58%.

The SEM analysis showed that the accumulation of 5-fluorourocil was localized on the surface of the spray-dried P(3HB) microparticles (Figure 3e). The revealed differences in the characteristics of the microparticles obtained using the two methods affected the rate of release of the drug in vitro. So, on the first day, the amount of the preparation released into the solution from microparticles, obtained using the emulsion method and the spray-drying method, was 5.1 ± 1.3% and 24.5 ± 1.5%, respectively. By the end of the experiment (700 h), the yield of 5-fluorourocil from the microparticles obtained using spray drying was four times higher, and amounted to 48.4 ± 1.6% (Figure 3e).

It is known that P(3HB) (and other representatives of PHAs) is not hydrolyzed in aqueous media, similar to polylactide, because PHA degradation (the destruction of the polymer to oligomers and monomers—3-hydroxybutyric acid) is biological, and occurs through cellular and humoral pathways. Therefore, the observed release of 5-fluorourocil was not associated with the degradation of the polymer, but occurred mainly as a result of the diffusion of the drug adsorbed on the surface of the particles, and also from the thickness of the particles through the pores.

The microparticles obtained using the emulsion method from P(3HB) had a relatively low total drug release. This may be related to the hydrophobicity of P(3HB), and the dense packing of polymer chains. Therefore, apparently, in the process of spraying the solution and the formation of drops during the transformation of microdroplets into microparticles, a significant part of the drug is clogged inside the particles. While the increase in the rate of release of 5-fluorourocil from the microparticles obtained by spray drying occurs due to the drug adsorbed on the surface of the particles. Therefore, apparently, the release of the drug in the first hours is related with the dissolution and leaching of free 5-fluoracil from the particle surface; and then (48–312 h) the outflow from the particles into the model medium was mainly realized as a result of diffusion of their volume of particles.

The results obtained indicate that the addition of PLGA and PEG to P(3HB) affected the properties of composite microparticles obtained by spray drying. Due to this, P(3HB) composites with polyethylene glycol and polylactide-co-glycolide in the ratio of components as P(3HB)/PEG75/25 and P(3HB)/PLGA 75/25, loaded with rifampicin and 5-fluorourocil were obtained by spray drying (Figure 3e–g). Microparticles from the homopolymer and composite P(3HB)/PLGA 75/25 loaded with rifampicin were a regular spherical shape with a smooth surface. Microparticles prepared from P(3HB)/PEG 75/25 with rifampicin had a rough surface with small pores. A similar surface structure was observed for drug-free P(3HB)/PEG 75/25microparticles (Figure 3e).

The loading of rifampicin particles was accompanied by the formation of agglomerates and the appearance of deformed particles. It is important to note that drug-loaded P(3HB)/PLGA microparticles had a mean diameter 3.8 μm and ζ potential from −49 mV. The morphology of composite microparticles was influenced not only by the chemical composition of the polymer base, but also by type of the drug. The microparticles obtained from the P(3HB)/PEG 75/25 composite, loaded with 5-fluorourocil, were of a regular spherical shape with fine pores over the entire surface of the particles (Figure 3f). It was found that 5-fluorourocil was predominantly on the surface of homopolymer microparticles, while the addition of drug to P(3HB)/PEG led to loading of 5-fluorourocil into the inner part of polymer microparticles.

This may be due to the fact that in the process of spray drying, the partial amorphization of substances occurs. The addition of surfactants, including polyethylene glycol, reduces amorphization [93]. The addition of a surfactant to solutions intended for spray drying leads to the formation of a smooth and spherical surface of the particles due to the dense packing of the molecules of the substances and a decrease in their mobility [94,95]. It can be assumed that the addition of polyethylene glycol to P3HB contributed to the fact that 5-fluorourocil was more uniformly distributed within the polymer chains and was densely packed during the drying process, providing high encapsulation efficiency and prolonged drug release.

The type of drug affected the efficacy of drug encapsulation. The encapsulation efficacy of 5-fluorourocil was lower when using P(3HB) and the P(3HB)/PEG 75/25 composite, amounting to about 57%. This figure for rifampicin was higher (70%). The identified particle differences affected the drug release in vitro. Accumulation of the drug concentration in the model medium was noted during the first 3 days. Further, the drug release slowed down. A higher release of rifampicin is characteristic of P(3HB)/PEG 75/25 microparticles, which amounted to 58.1 ± 1.7% by the end of the experiment. The release of rifampicin from particles obtained from P(3HB) and P(3HB)/PLGA75/25 was close and amounted to 49.7 ± 2.1% and 44.5 ± 1.7%, respectively.

A different picture was obtained for microparticles from P(3HB) and P(3HB)/PEG 75/25 loaded with 5-fluorourocil. The 5-FLU release from the homopolymer and composite P(3HB)/PEG microparticles was less than that of rifampicin. After 140 h, the drug release gradually decreased, and by the end of experiment the amount of drug released was 49.7% and 23% from the P(3HB) microparticles and composite P(3HB)/PEG microparticles, respectively.

The chemical nature of the drug determines the possibility of chemical interaction with the polymer carrier, which in turn can also affect the rate of drug release. In this work, a slight decrease the drug release rate of the anticancer drug compared to rifampicin a may have been due to the presence in the molecule of 5-fluorourocil reactive groups capable of forming covalent bond switch -COOH and –OH groups that are present in the polymer structure. It has been established that it is possible to regulate of the release rate of drugs from PHAs particles using polymers of various monomer compositions and also using PHAs composites with other materials.

### 2.4. Antibacterial and Antitumor Efficacy of Drug-Loaded PHA Microparticles

**Figure 4:**
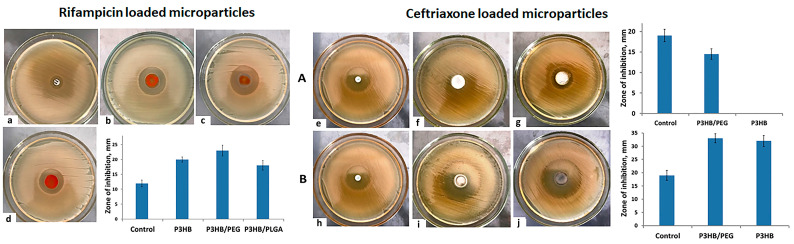
The antibacterial efficacy of drug-loaded microparticles in *E. coli* culture: rifampicin-loaded microparticles ((**a**) commercial disc, (**b**) P(3HB), (**c**) P(3HB)/PEG, (**d**) P(3HB)/PLGA) and ceftriaxone-loaded microparticles. (**A**) Microparticles prepared using solvent evaporation method. (**B**) Microparticles obtained using mini spray dryer ((**e**,**h**) commercial disc; (**f**,**i**) P(3HB)/PEG; (**g**,**j**) P(3HB)).

The drug efficacy of microparticles obtained using a P(3HB) composite base with polyethylene glycol P(3HB)/PEG and polylactide-co-glycolide P(3HB)/PLGA loaded with rifampicin or ceftriaxone was studied by the conventional disk diffusion method in a culture of an opportunistic test culture of *E. coli*. The results of bacterial inhibition are shown in Figure 4.

The drug efficacy of composite DDS was more influenced by the method of obtaining microparticles than by the chemical structure of the polymer. Thus, the maximum diameter of the *E. coli* inhibition zone is typical for composite microparticles from P(3HB)/PEG/75/25 obtained by spray drying and containing rifampicin −23 ± 1.8 mm. Microparticles with rifampicin, obtained from the composite P(3HB)/PLGA/75/25 and P(3HB) using a similar method, had close values for this indicator −18 ± 1.6 mm and 20 ± 0.9 mm, respectively. The average diameter of the *E. coli* inhibition zone when using microparticles made with a spray dryer and containing ceftriaxone was 31.5 ± 1.3 and 33 ± 1.5 mm. The sample prepared using the solvent evaporation method from composite microparticles with polyethylene glycol slightly inhibited the growth of *E. coli*, while the sample from pure P(3HB) did not inhibit the growth of *E. coli* at all (Figure 4A,B).

**Figure 5:**
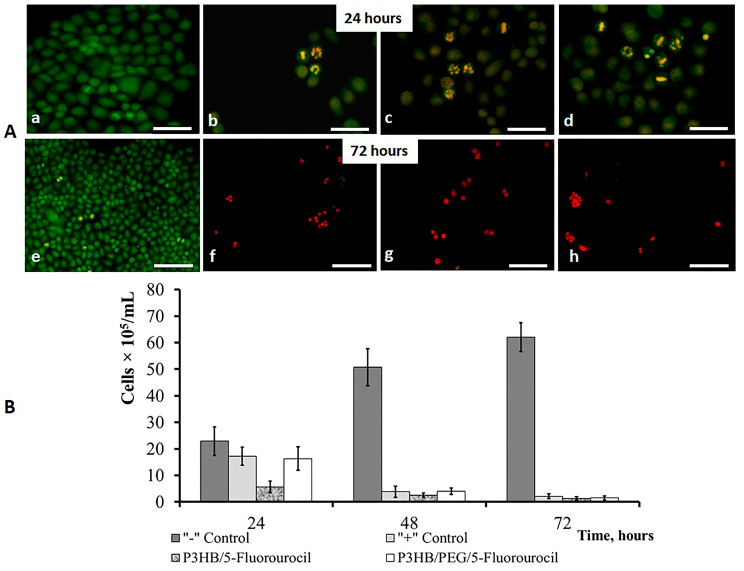
(**A**) Fluorescent staining of *HeLa* cells treated with 5-FLU ((**a**) “−” Control (intact cells); (**b**) “+” Control (free drug); (**c**)—P(3HB)/5-Fluorouracil; (**d**)—P(3HB)/PEG /5-fluorouracil; (**B**) MTT assay. Morphology of viable cells and nonviable cells when stained by acridine orange/ethidium bromide: viable cells stain uniformly green (**a**); early-apoptotic cells with intact plasma membranes appear light green, with condensed chromatin (**e**); apoptotic cells are stained bright green-orange because membrane deformation starts to occur, and EB can enter the cell (**b**–**d**); and late-apoptotic nonviable cells are stained bright orange or red because of the entry of ethidium bromide into these cells (**f**–**h**). The scale bar 10 μm (24 h) and 100 μm (72 h).

In the presented work, the drug efficacy of composite microparticles obtained from P(3HB)/PEG loaded with the antitumor drug 5-fluorourocil was evaluated in *HeLa* cell culture (Figure 5).

The greatest cytotoxic effect of 5-fluorouracil was detected after 24 h in the experimental group with P(3HB)/5-FLU. After 72 h, cytotoxic the effect of 5-fluorouracil loaded in P(3HB) microparticles was similar to the “+” control group (free drug).

Apparently, the high activity of 5-fluorouracil loaded into P(3HB) microparticles may be associated with the appearance of polymorphic changes in the drug during drying [96,97,98]. A similar effect was observed in the study with sucrose nanoparticles containing flurbiprofen. Flurbiprofen was also characterized by the transition to an amorphous state after passing through the spray drying [99].

Cytostatic drugs cause both apoptosis and cell necrosis. Cell death is assessed using differentiated fluorescent staining. Acridine orange stains intact cells. Ethidium bromide shows damaged and dead cells. Green color is characteristic of the nuclei of living cells. Green fragmented chromatin can be observed in cells with early apoptosis. The orange and red color is manifested by the presence of cells with late apoptosis [100,101]. In the “−”control group, the cells were oval and uniformly stained green. In the “+” control and experimental groups, we observed cells with apoptosis, including volume reduction and chromatin disruption. Cells with condensed chromatin by the type of apoptosis were observed a day later in all cells. After 72 h in the “−” control group, a few cells with destroyed chromatin were observed; in the experimental groups, there were an increase number of dead cells. The high ability of *HeLa* to proliferate, combined with the limited space of the culture plate, led to the appearance of apoptotic cells in the “−”control group.

It was noted that blank microparticles do not have a significant cytostatic effect on cell culture [78,102,103]. We have previously shown that PHA microparticles and films containing synthetic ozonide peroxide (OZ) inhibit cell growth and proliferation. The viability of HeLa cells when adding OZ-containing samples was 2–10%, compared to the control group (blank films and microparticles prepared from P3HB and P3HB/3HV), where the cell viability was at the level of 90% [104]. On the whole, in a culture of HeLa cells, the cytostatic efficacy of the 5-fluorouracil loaded in PHAs microparticles is shown.

## 3. Discussion

Drug delivery has attracted significant attention during the past decades owing to its extensive clinical needs. The big role in the processes of fabrication of DDSs in the form of particles is played by the technology of their manufacture and the material used as a base (matrix) [35]. With regard to PHAs, the emulsion method for obtaining microparticles is the most researched at present. The method consists of the dispersion and stabilization of the dispersed phase in an insoluble dispersion medium. The method principle is the packing of polymer chains in a liquid into spherical particles of various diameters [105,106,107]. In this case, the average size and size distribution of particles can depend on a number of factors, such as the type and concentration of surfactants, the concentration of the polymer solution, and the rate of mixing of the emulsion [108,109,110].

Using P(3HB) as an example, it was shown that an increase in the stirring speed led to a significant decrease in the average diameter of microparticles. Varying the density of the polymer solution and the method of dispersion of the emulsion also contributed to a decrease in the diameter of the microparticles. A similar possibility of obtaining particles of various sizes from P(3HB) using the emulsion method was noted by the authors of a series of works. In the work [105], using the emulsion method and mechanical mixing, microparticles from P(3HB) with a diameter of 5 to 100 μm were obtained. Modification of the emulsion method and the use of high-speed homogenizers led to the production of submicron-scale particles. Thus, Deepak et al. [111] obtained particles from P(3HB) with a diameter of 100–125 nm; the authors of another work [103] obtained smaller microparticles (about 50 nm) from the same type of PHAs. According to different authors, the average particle size obtained in a similar way from the P(3HB) ranged from 126 ± 155 and 199–250 nm (summarized in the review paper [32]).

Spray drying was another investigated method for the preparation of microparticles from P(3HB). This is a relatively simple and productive method for obtaining a microcarrier [61,66], but practically unexplored in relation to polymers of the PHA family. In a study from the available literature, microspheres from P(3HB) and poly-3-hydroxy-4-pentenoate (PHPE) loaded with paracetamol and phtalocianine (NzPC) used for photodynamic therapy were obtained and studied. In another work, microparticles were obtained from P(3HB) containing paclitaxel and their drug release was studied in vitro [74,75]. No other examples of obtaining microparticles from PHAs by spray drying have been found in the available literature.

The zeta potential is an important criterion for the stability of disperse systems with a liquid dispersion medium—suspensions, emulsions, and colloidal solutions. The magnitude of the ζ potential of microparticles depends on the chemical composition of the material, the pH, the temperature, the solvent, and the presence of electrolytes in the medium. The zeta potential can be used to predict the long-term stability of particles. For example, particles with zeta potentials larger than ±30 mV have excellent stability, whereas particles with zeta values between −5 mV and +5 mV will experience rapid agglomeration [112].

The different value of the ζ potentials of microparticles obtained using different methods is most likely due to the adsorption of surfactants on the particles, such as PVA. A similar effect was registered in the work [113]: the emulsion stabilizers strongly adhere to the particle surface.

P(3HB) is the most studied and available member of the PHA family [114]. However, its use is complicated by a number of negative aspects, including a high hydrophobicity and crystallinity, the closeness of its melting point and thermal degradation, which reduce its effectiveness, and the functional properties of polymer products, which have low mechanical strength and elasticity [115], age over time and are characterized by the lowest rates of biodegradation [116]. The copolymers PHAs, and composite materials with other biopolymers, are used to reduce these negative effects of P(3HB). The use of copolymer PHAs with different physicochemical properties will make it possible to influence the properties of the polymer base and the quality of DDS [31,32].

In this work, it is shown that the chemical composition of PHA affects the characteristics of microparticles. Thus, the ζ potential value for microparticles obtained from copolymers with different inclusions of 3HB, 3HV, and 3HHx monomers was more negative than for P(3HB). At the same time, the average diameter of microparticles slightly increased with an increase in the content of monomers in the copolymers P(3HB-co-4HB), P(3HB-co-3HV), P(3HB-co-3HHx). An analysis of the literature showed that the results obtained in the present work, on the effect of the composition of PHAs on the particle size, correspond to the prevailing ideas and the experimental results obtained. However, the published quantitative data differ significantly [31,32]. Thus, the average particle size from P(3HB-co-3HV) with the 3HV content of 5.0 and 11.0 mol.% varied in the range of 184–273 nm [103]. In another work [117], particles obtained by a similar method from similar copolymers, but with a large content of 3HV monomers (12.0 and 50.0 mol %) had close values of the average size (169.0–211.2 nm). Larger particles (up to 250; 300; 426 nm) from P(3HB-co-3HV) are described in the works [118,119].

The series of studies reported significantly different particle sizes prepared from the P(3HB-co-3HHx): from fine particles with an average diameter of 63.0 and 95.7 nm [120,121] to larger ones, from 200 to 273 nm [122,123], and very large (from 180 to 1500 nm) ones [124]. For DDS prepared from the less-crystalline and most elastic P(3HB-co-4HB) copolymers, a few papers have described particles with similar average sizes, on the order of 150 nm [125,126].

An analysis of the literature indicates that the drug release may vary depending on the physicochemical properties of the polymeric materials of the carrier, the chemical nature of the drug, and environmental parameters (pH, ionic strength of the solution, the presence/absence of enzymes, etc.) [32,127,128].

In this work, it was shown that the drug release rate depends on the monomeric composition of polymer, the drug content and microparticle manufacturing method. Also, the literature data indicate the possibility of controlling the kinetics of drug release from DDS by changing the chemical composition of PHAs [32]. The authors of [129] described the dynamics of progesterone release from microparticles obtained from a P(3HB-co-3HV) copolymer with different contents of 3HVmonomers. It has been shown that with an increase in the content of 3HV monomers in the copolymer from 9 to 24 mol.%, the release of progesterone increases due to a change in the surface and the formation of pores. Conversely, in the work [103] it was shown that, regardless of the content of the 3HV monomer P(3HB-co-3HV), nanoparticles loaded with ellipticin did not demonstrate statistical differences and caused a stronger inhibition of cancer cells (almost twice) compared to free ellipticin. Less-mastered and more-difficult-to-synthesize PHA copolymers containing 3HHx or 3-hydroxyoctanoate monomers with reduced crystallinity are more suitable for use as a carrier of drug carriers. Using the example of a number of works, the effectiveness of these types of PHAs for the design of DDS was shown. Thus, the release of rhodamine isothiocyanate from microparticles derived from the P(3HB-co-3HHx) copolymers was much more active compared to particles from P(3HB) microparticles [130].

In another study, P(3HB-co-3HHx) nanoparticles were obtained by the emulsification–solvent evaporation method [123], which had an average diameter of 200 nm and were loaded with rapamycin, providing a smooth and prolonged release of the drug for 10 days. The authors of these and other works believe that the hydrophobicity of PHAs slows down the degradation of nanoparticles, which prolongs the release of drugs.

Available publications indicate the possibility of obtaining long-acting drugs with antibacterial and antitumor activity using only the emulsion method of preparation for PHA-based microparticles. For example, copolymer microparticles from P(3HB-co-HHx) loaded with curcumin and conjugated with the targeting ligand exhibited high apoptotic activity against breast cancer cells [122]. A high antitumor activity of P(3HB-co-3HHx) nanoparticles with folic acid was demonstrated in *HeLa* cell culture [124]. The P(3HV-co-4HB)-bmPEG copolymer PHA–polyethylene glycol particles enhanced the apoptotic activity of encapsulated cisplatin on the DU145 prostate cancer cell line [131]. The drug efficiency of P(3HB-co-PEG) nanoparticles loaded with an antisense oligonucleotide were effective in suppressing cancer cell line A549 [132]. Doxorubicin loaded in P(HB-co-HO) copolymer nanoparticles had higher cytotoxicity against *HeLa* compared to the free drug. The authors showed that the death of *HeLa* cells under the action of the obtained nanoparticles increased by a factor of 30 when an active targeting ligand was added [130]. In one of the last works [127], P(3HB-co-3HV) microspheres loaded with doxorubicin and radioactive agent (^153^Sm) was successfully developed for transarterial chemoembolization and transarterial radioembolization. Obtaining microspheres had a pronounced cytotoxic effect in HepG2 cell line.

In this work, the drug efficacy of composite microparticles loaded with antibiotics and cytostatics has been demonstrated in cell cultures (*E. coli* and *HeLa*, respectively). In general, it was shown that P(3HB)/PEG composite microparticles containing ceftriaxone and obtained by spray drying had a more pronounced antibacterial effect on the *E. coli* culture. Similar results have been described in the work of colleagues. Weak inhibition of the growth of microorganisms by ceftriaxone deposited in lipospheres with PEG was explained by the authors by the fact that the concentration of the drug contained in the lipospheres was insufficient for inhibition. In the work [132], P(3HB-co-3HV) microparticles prepared using the emulsion method containing ceftiofur also showed the slight inhibition of *E. coli*.

The cytostatic effect of deposited 5-fluorourocil in microparticles obtained by spray drying from P(3HB) and P(3HB)/PEG was generally comparable to the effect of the free drug on a *HeLa* cell culture. At the same time, the manifested prolonged cytostatic effect is an indication that the deposited 5-fluorourocil in P(3HB) and P(3HB)/PEG microparticles are stable and are not inactivated during spray drying.

The results indicate a high potential of biocompatible and biodegradable polymers of the PHA family for the design of modern drug delivery systems. Such comprehensive studies, combining the issues of the influence of manufacturing methods and the chemical composition of the polymer, on the characteristics of PHAs microparticles and the drug release rate, including the assessment of their drug efficacy, have not been identified in the available literature. Therefore, the results obtained can be a basis for further research, in particular, when using the less-studied method of spray drying for the development of DDSs based on PHAs.

## 4. Materials and Methods

### 4.1. Materials

High-purity PHA specimens were produced in the IBPSB RAS using the microbial fermentation process using various precursors (potassium valerate, potassium hexanoate, ɛ-caprolactone) and carbon source (sugars, fat-containing wastes) [133] (Table 2). The PHA concentrations and compositions were analyzed using gas chromatograph–mass spectrometry (Hewlett Packard, USA). The technology for the synthesis of PHAs of various chemical compositions, and methods for studying the physicochemical properties (molecular weight (M_w_, M_n_, D) and temperature (T_melt_, T_degr_) characteristics, degree of crystallinity (C_x_)) have been described in detail in earlier works [134,135,136].

As the drugs for encapsulation into the microparticles, we used the antibiotics ceftriaxone (Farm-Center, Russia) and rifampicin (ZAO BRYNTSALOV-A, Moscow, Russia) and the antitumor drugs Doxorubicin-LENS (Farm-Center, Russia), and 5-fluorouracil (Sigma-Aldrich, St. Louis, MI, USA) (Table 3).

The following materials were used in this work: polyvinyl alcohol (PVA) was purchased from Sigma-Aldrich (St. Louis, MI, USA), polyethylene glycol (PEG, Mw 35 kDa) from Sigma-Aldrich (St. Louis, MI, USA). PLGA (Mw 19 kDa) was bought from ACROS (Stuttgart, Germany). Sodium borohydride was provided by Merck (Darmstadt, Germany). Furthermore, dichloromethane (DCM) and methyl alcohol were provided from ChemProm (Moscow, Russia), and distilled water (MilliQ). All the used chemicals were of analytical grade. Dulbecco’s modified Eagle’s medium (DMEM) was obtained from Gibco (Grand Island, NY, USA). Mueller–Hinton medium was purchased BioRad (Marnes-la-Coquette, France). 3-(4,5-dimethylthiazol-2-yl)-2,5-diphenyltetrazolium bromide (MTT) and fluorescein isothiocyanate (FITC) were purchased from Sigma-Aldrich (St. Louis, MI, USA). Acridine orange was bought in Biolot (Moscow, Russia).

### 4.2. Methods of Preparation of PHAs Microparticles

**Figure 6:**
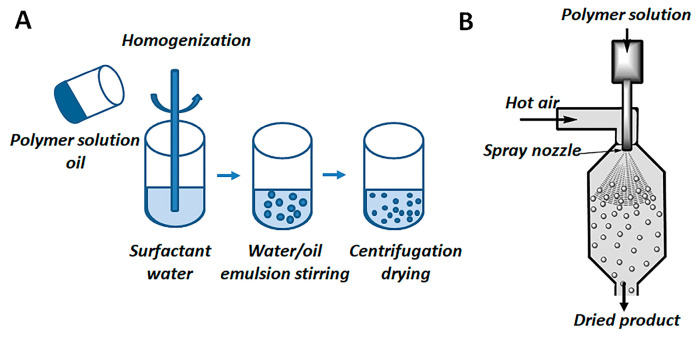
Schematic representation of methods for obtaining PHA microparticles: emulsion method (**A**), and spray drying (**B**).

PHA microparticles were obtained using two manufacturing methods—the emulsion method and the spray-drying method. A schematic representation of these methods is shown in Figure 6.

When testing the production of microparticles using the emulsion method, the emulsion stirring rate, the emulsion dispersion method, and the concentration of the polymer solution and the monomer composition of the PHAs were varied. In the case of obtaining microparticles using spray drying, the flow rate of the solution and the temperature at the inlet to the system were changed.

Microparticles were obtained via solvent evaporation from the double (water/oil) and triple (water/oil/water) emulsions. The double emulsion contained a 1% or 2% or 4% (*w*/*v*) solution of polymer in DCM and 0.5% (*w*/*v*) solution of PVA. The triple emulsions were prepared through the addition of an aqueous solution of drug to the polymer solution. Polymer emulsions were mixed with a Heipolph RZR overhead-drive three-blade stirrer (Germany) at various stirring rates (500–1000 rpm), an IKA Ultra-Turrax T25 high-speed homogenizer (Germany) (15,000 and 24,000 rpm), and a Sonicator S3000 ultrasonic generator (USA). The ultrasound power was varied from 12 to 20 V, and the treatment lasted between 60 and 300 s. The microparticles were centrifuged at a speed of 10,000 rpm (14,087 *g*) for 5 min (Hanil Combi 514R, South Korea) washed five times with distilled water, and dried in a LS-500 lyophilizer (Russia).

Other sample microparticles were prepared using spray drying with a Buchi B-290 Spray dryer (BUCHIL a laboratory Equipment, Switzerland). The feed solution of PHAs P(3HB), P(3HB)/PEG (75:25) or P(3HB)/PLGA (75:25) in DCM (1% (*w*/*v*)) was used. The inlet temperature was set to 75–95 °C, and the feed rate was 1.5–5 mL/min. The co-current drying air had a flow rate of 0.6 m^3^/min, and the atomizing air was supplied with a pressure of 1.25 bar. The yield of microparticles was calculated based on the weight of microparticles and the weight of the polymers used to prepare the microparticles.

### 4.3. Characterization of Microparticles

#### 4.3.1. Particle Size, Size Distribution and ζ Potential

The mean diameter of the microparticles over 10 μm was determined using a system for quantitative particle analysis—Flow Cam (Fluid Imaging, Scarborough, ME, USA). The size distribution of microparticles less than 10 μm and the value of the ζ potential were determined using the DLS method on a particle size analyzer Zetasizer Nano ZS (Malvern Panalytical, Worcestershire, UK).

#### 4.3.2. Microscopic Analysis

The surface morphology of the microparticles was investigated using scanning electron microscopy FEI Company Quanta 20 (Hillsboro, OR, USA) and S-5500 electron microscopy (Hitachi, Tokyo, Japan). The samples were coated with platinum by a putter coater K550X (Emitech, Quorum Technologies Ltd., Lewes, UK).

#### 4.3.3. Loading of Drugs and Encapsulation Efficiency

To obtain drug-loaded microparticles, the emulsion method and the spray-drying method were used. In the case of a double emulsion, doxorubicin (5% of the mass of the polymer carrier) was dissolved in DCM, and then a weighed portion of the polymer was added to the resulting solution. The 5-fluorouracil (5% of the mass of the polymer carrier) was preliminary dissolved in methanol and mixed with a solution of the polymer.

To obtain the triple emulsion, the polymer was dissolved in DCM, and the solution of the ceftriaxone (from 1 to 10% (*w*/*w*)) was added. After that, solution was homogenized using ultrasound (1 min, 12 W). Then, obtained emulsions were homogenized using an IKA Ultra-Turrax T25 digital high-speed homogenizer, Germany, at 24,000 rpm). The centrifugation and washing conditions were similar to as described earlier.

Spray-dried microparticles loaded with 5-fluorouracil (5% (*w*/*w*)) were prepared using a 1% (*w*/*v*) solution P(3HB) dissolved in DCM and 5-fluorouracil dissolved in methanol. Microparticle-loaded rifampicine was prepared using the spray-drying process. For this drug, 5 % (*w*/*w*) and polymer P(3HB) or P(3HB)/PEG (75:25) or P(3HB)/PLGA (75:25) was dissolved in DCM and sprayed at an inlet temperature of 75 °C, with a feed pump speed 1.5 mL/min.

The quantity of the drug loaded in the PHA microparticles was measured on a UV–Vis spectrophotometer Genesys 10S (Thermo Scientific, Waltham, WA, USA). The absorbance of doxorubicin was 405 nm, ceftriaxone 240 nm, 5-fluorouracil 265 nm, and rifampicin 475 nm.

The encapsulation efficiency of the different drugs within PHA microparticles was calculated by dividing the encapsulated drug amount by the total amount of the drug introduced in the microparticles.

#### 4.3.4. In Vitro Release Studies

The drug-loaded PHA microparticles were used in the in vitro release studies. For this, the PHA microparticles were sterilized using UV radiation and introduced into sterile tubes, containing 2 mL phosphate-buffered saline (PBS, pH 7.4); the tubes were incubated in a thermostat within 28 days. The withdrawn samples were centrifuged for 10 min at 10,000 rpm (9408 *g*) (Eppendorf 5418R) and the drug concentration was measured using a Genesys 10S UV–Vis spectrophotometer (Thermo Scientific, Waltham, WA, USA). Triplicate measurements were performed during every analysis.

### 4.4. Antimicrobial and Antitumor Efficacy of Drug-Loaded PHA Microparticles

The disc diffusion method was used for assay of the antibacterial activity of the antibiotic-loaded microparticles. The antimicrobial properties of the microparticles were tested against the Gram-negative bacteria *Escherichia coli* [137]. In brief, the disks of antibacterial drugs (ceftriaxone and rifampicin) were used as the control (the amount of the drug was 0.03 mg). The amount of ceftriaxone and rifampicin loaded in the PHA microparticles was 0.3 mg. The samples were incubated in a thermostat at 37 °C. After 24 h, the diameters of the zones of inhibition of bacterial growth were measured.

The antitumor efficacy of the 5-fluorouracil loaded in the P(3HB) and P(3HB)/PEG microparticles was examined in a culture of human cervical carcinoma cells (*HeLa*) at the concentration of 5 × 10^5^ cells/mL. When incorporating the microparticles in the cell culture in the form of a suspension concentration, the encapsulated 5-fluorouracil was 0.6 mg/mL. The cultivation was carried out according to the method described earlier [138]. The cytotoxicity of the 5-fluorouracil loaded in P(3HB) and P(3HB)/PEG microparticles against *HeLa* cells was measured using the MTT assay. The *HeLa* cells treated with 5-fluorouracil loaded in the PHA microparticles were stained using acridine orange and ethidium bromide as described in the protocol [139], and their qualitative reaction (live/dead cells) was performed using a Leica DM6000B fluorescence microscope (Germany).

### 4.5. Statistical Analysis

The statistical processing of the results was carried out using the standard Microsoft Excel. Student’s *t*-test was used to analyze the statistical significance of the experimental results. Experimental data were expressed as arithmetic means ± SD (*n* = 3 for all experiments), and *p* < 0.05 was considered a significant difference.

## 5. Conclusions

Using natural polymers of PHAs as a degradable base, DDSs in the form of microparticles, including those loaded with drugs, were designed and studied. To obtain microparticles, the emulsion method and the spray-drying method and four types of PHAs were used, differing in their sets and ratios of monomers and properties (degree of crystallinity, molecular weight and temperature characteristics): P(3HB) and three types of copolymers, P(3HB-co-3HV), P(3HB-co-4HB), P(3HB-co-3HHx). The characteristics of microparticles (morphology, size distribution, ζ potential) depending on the method of preparation and monomer composition of PHAs were studied. By changing the conditions of preparation and the type of PHAs, good-quality particles, with an average diameter from 0.8 to 65.0 μm and ζ potential values from −18 to −50 mV, were obtained. The drugs’ releases in vitro, depending on the particle size, load value and type of drugs, were studied. The drug efficacy of the composite microparticles loaded with antibiotics and cytostatics was demonstrated in cell cultures (*E. coli* and *HeLa*, respectively). The results indicate a high potential of biocompatible and biodegradable polymers of the PHA family for the design of modern and efficient drug storage and delivery systems.

## Figures and Tables

**Table 1:** Studied properties of P(3HB) microparticles.

**Concentration** **of P(3HB), %**	**Dispersion Technique**	**Average Diameter,** **μm**	**ζ Potential, ** **mV**	**Yield of Microparticles,** **%**
Emulsion method				
1.0	Hh *	1.2 ± 0.09	−16.2 ± 0.49	73.8 ± 4.6
2.0	Hh *	1.58 ± 0.15	−15 ± 0.28	78.5 ± 3.8
4.0	Hh *	2.32 ± 0.3	−21.6 ± 0.7	78.7 ± 4.3
2.0	Us 12 *	2.5 ± 0.14	−23 ± 0.31	56 ± 4.2
2.0	Us 20 *	1.2 ± 0.08	−19 ± 0.25	61.5 ± 3.5
		Spray drying		
**Temperature, °C**	**Solution Feed Rate, mL/min**	**Average Diameter,** **μm**	**ζ Potential,** **mV**	**Yield Microparticles,** **%**
75	1.5	3.4 ± 0.6	−45 ± 0.5	83.5
75	3.2	3.8 ± 0.5	−44.5 ± 1.1	71
75	5.0	5.5± 0.5	−43.2± 1.2	85
85	1.5	5.7 ± 0.5	−47.1 ± 0.4	78
85	3.2	5.6 ± 0.6	−42.3 ± 0.6	90
85	5.0	5.2 ± 0.3	−49 ± 3.4	45
95	1.5	4.2 ± 0.3	−41.6 ± 0.8	75
95	3.2	7.0 ± 0.48	−47.4 ± 0.4	71
95	5.0	6.2 ± 0.5	−50.8 ± 0.5	75

Hh *—high-speed homogenization (15,000 rpm). Us 12 *—ultrasound 12 V. Us 20 *—ultrasound 20 V.

**Table 2:** Biodegradable PHAs of different chemical compositions used to prepare microparticles.

PHA Composition, mol.%	Structural Formula	M_w_, kDa	M_n_, kDa	D	C_x_, %	T_melt_, °C	T_degr_, °C
		**P(3HB)**					
100.0	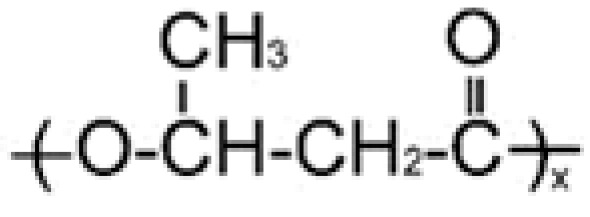	1200	710	1.69	76	169	272
**P(3HB/3HV)**							
93.2/6.8	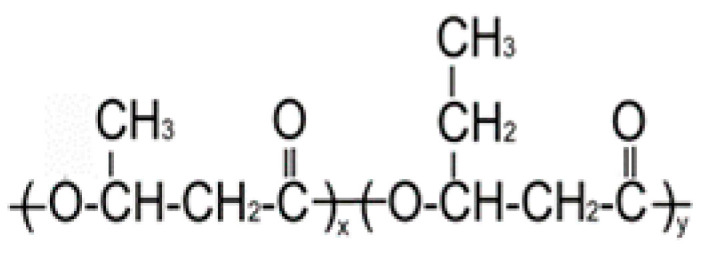	890	466	1.91	60	162	264
67.2/32.8		398	115	3.46	54	160	259
**P(3HB/4HHx)**							
93.0/7.0	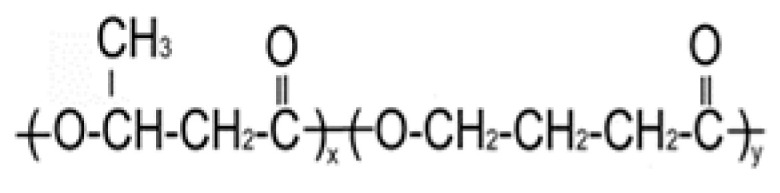	410	140	2.93	56	162	258
86.4/13.6		390	130	3.00	49	160	264
		**P(3HB/4HB)**					
93.9/6.1	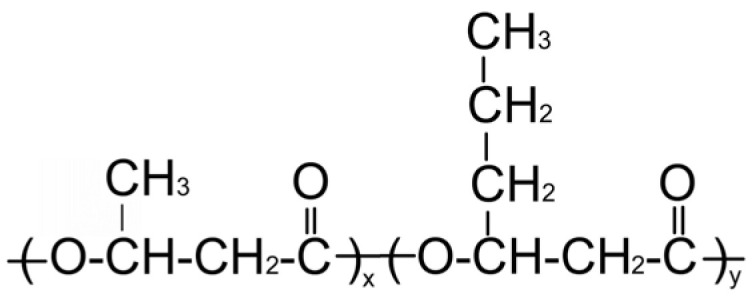	416	142	2.93	52	160	254
84.0/16.0		520	168	3.10	41	154	260

**Table 3:** Drugs encapsulated in polymer microparticles and their mechanism of action.

Drug	Structural Formula	Mechanism of Action
Ceftriaxone	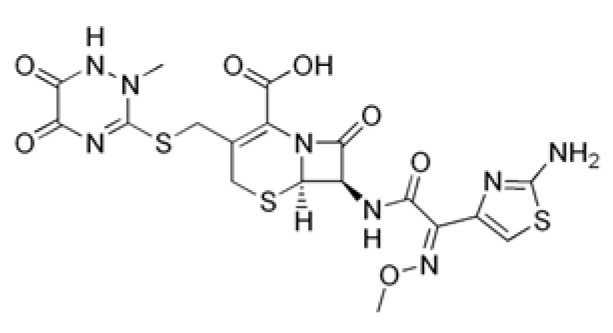	Inhibition of bacterial cell wall synthesis through acetylation of membrane-bound transpeptidases with further disruption of cell wall peptidoglycan synthesis.
Rifampicin	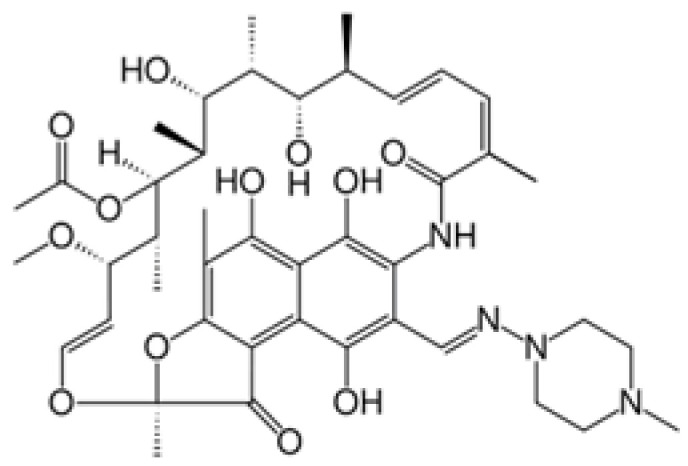	Violates RNA synthesis in a bacterial cell: binds to the beta subunit of DNA-dependent RNA polymerase, preventing its attachment to DNA, and inhibits RNA transcription.
Doxorubicin	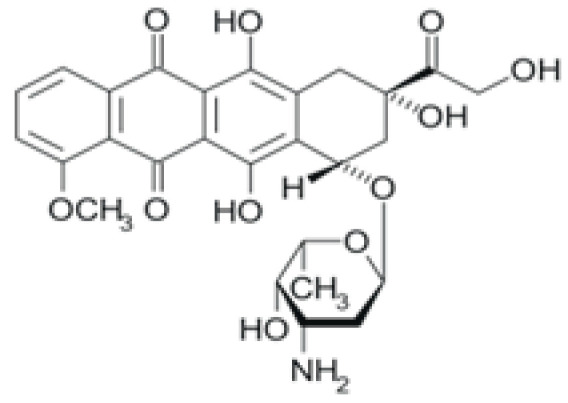	Suppression of the synthesis of nucleic acids, followed by the formation of free radicals and effect on cell membranes.
5-Fluorourocil	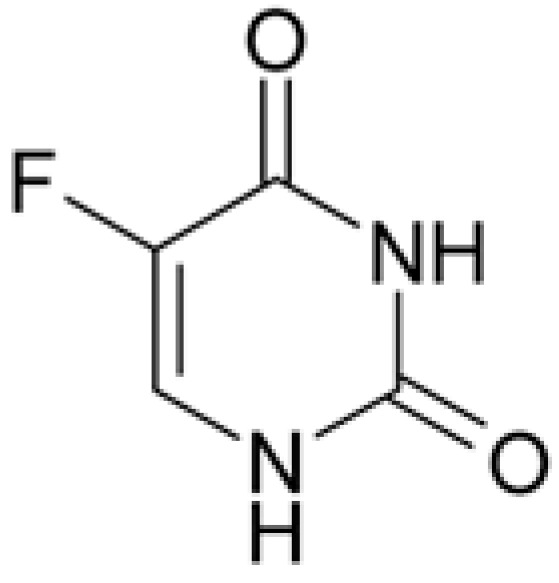	Inhibits the process of cell division by blocking DNA synthesis (due to inhibition of thymidylate synthetase enzyme activity) and the formation of structurally imperfect RNA (due to the introduction of fluorouracil into its structure).

## Data Availability

All data are available in the paper.
